# Increased and continuous coronary arterial flow was induced by LV uncoupling condition using combined treatment of a microaxial heart pump and venoarterial extracorporeal membrane oxygenation

**DOI:** 10.14814/phy2.15084

**Published:** 2021-10-21

**Authors:** Motoko Kametani, Masahiro Yamada, Yoko Horibata, Tomohiro Sakamoto, Takashi Unoki

**Affiliations:** ^1^ Division of Cardiovascular Medicine Saiseikai Kumamoto Hospital Cardiovascular center Kumamoto City Kumamoto Japan

**Keywords:** coronary artery, ECPELLA, LV uncoupling, refractory cardiogenic shock

## Abstract

An emerging therapeutic modality, ECPELLA, which combines a transvalvular microaxial left ventricular (LV) assist device, Impella, and venoarterial membrane oxygenation (VA‐ECMO), has been applied for patients with refractory cardiogenic shock. During ECPELLA support, VA‐ECMO increases the LV load, whereas the Impella reduces the LV load. Studies reported that coronary perfusion is influenced by LV unloading conditions, and the effective degree of LV unloading to increase the coronary perfusion on ECPELLA support remains to be determined. Here, we reported a cardiogenic shock case whose coronary arterial flow was assessed by transesophageal echocardiography during ECPELLA support. The left anterior descending coronary artery (LAD) peak blood flow velocity and the velocity time integral (VTI) were not significantly increased when blood was ejected from the LV (partial LV unloading). When the LV blood ejection was completely bypassed by Impella confirmed by non‐pulsatile aortic pressure with significantly reduced LV pressure with no aortic valve opening (LV uncoupling: no blood ejection from the LV), both peak velocity and VTI of the LAD were markedly increased and the blood flow became continuous throughout the cardiac cycle. Our case suggests that the coronary arterial flow in the injured myocardium is sensitive to degrees of LV unloading on ECPELLA support.

## Graphical abstract

Continuous coronary blood flow was observed when the left ventricle (LV) is uncoupled from the systemic circulation by Impella pump support where the LV pressure never exceeds the aortic pressure during the entire cardiac cycle.

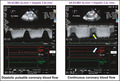

## 1 INTRODUCTION

An emerging lifesaving therapeutic modality, called ECPELLA, which combines two mechanical circulatory supports, a transvalvular microaxial Impella pump (Abiomed Inc. Danvers, MA) and venoarterial extracorporeal membrane oxygenation (VA‐ECMO) (Tongers et al., 2020). The ECPELLA enables simultaneous end‐organ perfusion and the LV unloading in which VA‐ECMO increases the left ventricular (LV) mechanical load, whereas Impella reduces the LV load (Hamanaka et al., 2020). The VA‐ECMO withdraws and oxygenizes the systemic venous blood, and returns it into the descending aorta by which the systemic venous return is partially reduced and LV afterload is increased. The transvalvular microaxial pump Impella directly pumps out the blood from the LV resulting in LV preload reduction with increase in total circulatory flow, which also increases the systemic venous return. The increased LV afterload by VA‐ECMO can be adjusted by Impella and increased systemic venous return can be adjusted by VA‐ECMO vice versa. In addition, patients who require ECPELLA support have severe right and/or left heart dysfunction and the cardiac function can be very sensitive to changes in circulatory blood flow. Thus, it is important to determine adequate circulatory blood flow balance between pulmonary and systemic circulations. In this unique hemodynamic condition, the coronary perfusion should be affected, however, the coronary flow effect on ECPELLA support has not yet been well documented.

Previous studies showed that coronary perfusion is increased by the LV unloading (Hamanaka et al., 2020; Remmelink et al., 2007). However, it remains to be investigated whether ECPELLA can increase coronary arterial flow. Here, we reported a case on ECPELLA support in which the coronary arterial flow was markedly increased only at the LV uncoupling condition.

## 2 CASE

We obtained the consent from the authorized family member at the emergency room. This study complied with the Declaration of Helsinki and was approved by the Institutional Ethics Committee (approval numbers #875 “ECPELLA/ECMO + IABP observational research,” and #925 “Left ventricular pressure measurement in patients on ECPELLA support,” Saiseikai Kumamoto Hospital Ethics Committee).

A 62‐year‐old male suffered from refractory cardiogenic shock due to lethal ventricular arrhythmias after successful percutaneous coronary intervention for old anteroseptal myocardial infarction. The patient underwent ECPELLA support, and the proximal left anterior descending coronary artery (LAD) flow was measured at different VA‐ECMO and Impella support conditions using the transesophageal echocardiogram.

**FIGURE 1:**
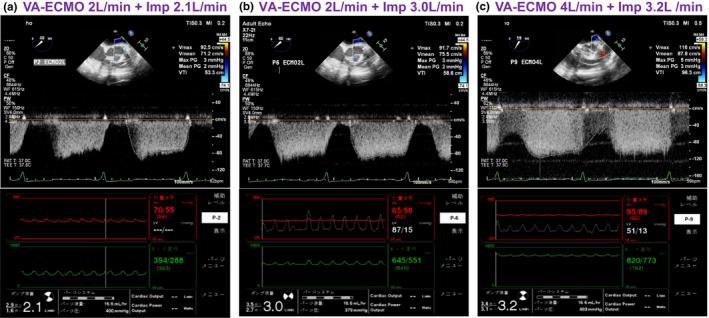
Left coronary anterior descending artery flow, the aortic pressure, and the Impella motor current waveforms on the Impella controller at different hemodynamic support and LV unloading conditions. Panel (a) shows the pulsed Doppler blood flow of the left anterior descending artery (LAD), and the corresponding aortic pressure and Impella motor current waveforms on 2.0 L/min VA‐ECMO and 2.1 L/min Impella support. Panel (b) shows LAD flow, the aortic pressure, estimated LV pressure, and Impella motor current waveforms on 2.0 L/min VA‐ECMO and 3.0 L/min Impella support. Panel (c) shows LAD flow, the aortic pressure, estimated LV pressure, and Impella motor current waveforms on 4.0 L/min VA‐ECMO and 3.2 L/min Impella support. The LAD flow was not significantly increased when blood was ejected from the LV [panels (a) and (b), partial LV support] despite mean Impella support was increased from 2.0 to 3.0 L/min. When the aortic pressure became non‐pulsatile and the estimated systolic LV pressure never reached the aortic pressure throughout the entire cardiac cycle (estimated peak aortic and the LV pressures were 95 and 51 mmHg, respectively) and the blood ejection from the LV was fully bypassed by Impella (LV uncoupling), LAD flow became continuous, and the peak flow velocity and corresponding VTI were markedly increased [panel (c)]. It is noted that the LAD internal diameter measured by the B‐mode echocardiogram was not changed at different VA‐ECMO and Impella support conditions (4.2 mm, data not shown)

When VA‐ECMO support was set at 2.0 L/min and the mean Impella support levels were increased from 2.1 to 3.0 L/min, LAD flow was detected during the diastolic phase and the peak LAD flow velocity (92.5–91.7 cm/s) and the velocity time integral (VTI: 53.3–58.6 cm) were not significantly changed. The estimated LV pressure on the Impella controller was same or exceeded the aortic pressure at the early systolic phase, and the aortic valve motion on the echocardiogram indicated blood was ejected from the LV (Figure 1, panels a,b, and Video S1). When VA‐ECMO and mean Impella flows were, respectively, increased to 4.0 and 3.2 L/min, the Impella controller displayed the estimated LV pressure was below the aortic pressure throughout the cardiac cycle and the echocardiogram confirmed no aortic valve opening (LV uncoupling condition, Video S1) (Saku et al., 2018). At the LV uncoupling condition, LAD flow became continuous throughout the entire cardiac cycle, and both peak velocity and the VTI were markedly increased to 116 and 96.3 cm, respectively (Figure 1, panel c and Video S1). We maintained the LV uncoupling condition for the first 48 h until the LV function was being recovered and then, the VA‐ECMO and Impella were successfully weaned off on day 3 and day 8, respectively.

## 3 DISCUSSION

Coronary artery flow is influenced by pressure gradient between given aortic pressure and LV pressure, myocardial microcirculatory pressure, and ventricular contraction (Alqarqaz et al., 2018; Davies et al., 2006; Hamanaka et al., 2020). Davies et al. (2006) analyzed coronary flow waves in which the coronary arterial flow was driven by forward pushing flow wave by LV ejection and a backward propagating suction flow wave by rapid relief of myocardial microcirculatory compression at the early diastolic phase. Other studies showed that the LV unloading induced by Impella reduced the LV end‐diastolic pressure and increased coronary perfusion pressure and flow (Alqarqaz et al., 2018; Hamanaka et al., 2020; Remmelink et al., 2007).

In the current case, we simultaneously monitored Impella flows, estimated LV pressure on the Impella controller, and the aortic valve opening (Video S1) by echocardiography. When the aortic valve was opened during the systolic phase on ECPELLA support (partial LV support, panel b), LAD flow was pulsatile with the peak coronary flow velocity at the early diastolic phase. The VTI did not markedly increase even when Impella flow was increased, and the estimated LV pressure reached the aortic pressure during the systolic phase (aortic valve was opened by LV blood ejection). In contrast, both peak coronary artery flow velocity and the VTI were markedly increased with continuous coronary flow pattern throughout the cardiac cycle when the LV was uncoupled from the systemic circulation (LV pressure <the aortic pressure throughout the cardiac cycle, panel c). At the LV uncoupling condition, the aortic valve was consistently closed throughout the entire cardiac cycle. The systemic circulation was fully supported by VA‐ECMO and Impella with non‐pulsatile arterial flow. According to the manufacture's instruction and a previous study, the positive Impella outlet–inlet pressure gradient at given maximum and minimum Impella flows shown in the panel c indicates LV uncoupling condition where LV pressure was consistently lower than the aortic pressure throughout the cardiac cycle (Chang et al., 2018).

In order to achieve LV uncoupling condition, we increased total mechanical circulatory support (VA‐ECMO 4.0 L/min + Impella 3.2 L/min) (panel c), and the aortic pressure also markedly increased compared to the partial LV support (panels a,b). It is noted that the LAD diameter measured by B‐mode ultrasound image was not changed (4.2 mm, data not shown). We speculate that full ECPELLA support with LV uncoupling condition increased the aortic pressure in a non‐pulsatile manner with markedly reduced LV pressure resulting in increased coronary perfusion pressure. Although both LV forward coronary flow wave and backward suction flow wave were reduced under the LV uncoupling condition, the elevated aortic pressure and the consistently reduced LV pressure throughout the cardiac cycle with reduced myocardial microcirculatory pressure result in increased peak flow velocity and the VTI with continuous coronary flow pattern (panel c). This coronary flow pattern could be only achieved by ECPELLA with LV uncoupling condition.

In the animal model of ischemic myocardium, reduction of ischemic myocardial size was associated with degree of the LV unloading in which the LV uncoupling condition showed significantly less myocardial damage (Saku et al., 2018). Therefore, degree of LV unloading on ECPELLA support could be very important for adequate coronary perfusion of the damaged myocardium. Further studies are necessary to determine the effective degree of the LV unloading on ECPELLA support.

## CONFLICT OF INTEREST

All authors have no conflict of interest to disclose.

## AUTHORS’ CONTRIBUTIONS

Makoto Kametani (MK): Research design, data collection, data analyses and interpretation, and manuscript writing. Masahiro Yamada (MY): Research design and data collection. Yoko Horibata (TH): Research design and data collection. Tomohiro Sakamoto (TS): Research design, data interpretation, and manuscript writing. Takashi Unoki (TU): Corresponding author, research design, data collection, data analyses and interpretation, and manuscript writing.

## Supporting information



Video S1
